# Synthesis of 1,3-*cis*-disubstituted sterically encumbered imidazolidinone organocatalysts

**DOI:** 10.3762/bjoc.13.254

**Published:** 2017-12-01

**Authors:** Jan Wallbaum, Daniel B Werz

**Affiliations:** 1 Institut für Organische Chemie, Technische Universität Braunschweig, Hagenring 30, 38106 Braunschweig, Germany

**Keywords:** imidazolidinone, MacMillan catalyst, organocatalysis

## Abstract

A variety of novel imidazolidinone-based organocatalysts with bulky substituents were synthesized under mild reaction conditions starting from easily accessible substrates. Different natural and unnatural amino acid methyl amides were cyclized with aromatic carbaldehydes to yield two diastereomeric MacMillan-type catalysts. Special emphasis was put on bulky residues such as mesityl and pyrene moieties.

## Introduction

**Scheme 1:**
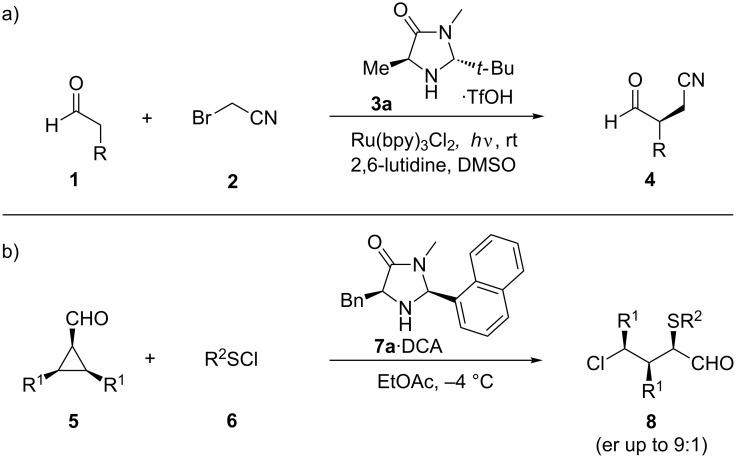
a) MacMillan’s enantioselective α-alkylation of aldehydes. b) Our enantioselective 1,3-chlorosulfenation of *meso*-cyclopropyl carbaldehydes.

Organocatalytic iminium and enamine activation has attracted organic chemists for more than one century [1]. Until today a wide and constantly increasing number of different organocatalytic transformations with various substrates have been accomplished [2–7]. Initially, proline and proline-derived catalysts have been widely used in asymmetric iminium and enamine organocatalysis [8–12]. Since the beginning of the 21th century imidazolidinone-based organocatalysts developed by MacMillan and co-workers, which are easily accessible from amino acids, are widely used in these kinds of reactions [13–18]. More recent examples demonstrate the applicability in various reactions like diastereoselective α-fluorination [19], total syntheses [20–21], cross-dehydrogenative couplings [22], selectivity-reversed Friedel–Crafts alkylation [23] and in combination with photoredox catalysis (Scheme 1a) [24].

The enantioselective α-alkylation was achieved by merging the common photoredox catalyst Ru(bpy)_3_Cl_2_ with imidazolidinone catalyst **3a**·TfOH, controlling the stereochemistry of the radical addition via an intermediate enamine complex.

Scheme 1b shows our regio-, diastereo- and enantioselective 1,3-chlorosulfenation of *meso*-cyclopropyl carbaldehydes employing a newly designed organocatalyst **7a**·DCA for chiral induction [25]. In the course of these studies we prepared a variety of imidazolidinone organocatalysts with rather bulky substituents. In this paper we report on these MacMillan-type catalysts which will be of great value for the screening of further transformations based on iminium–enamine mechanisms.

## Results and Discussion

In order to screen a certain transformation by using a variety of organocatalysts with subtle differences in the substitution pattern a modular approach for their preparation is highly desirable. A simple retrosynthetic cut of the five-membered ring delivers an amino acid methyl amide and a carbaldehyde as starting materials. Chirality is commonly introduced by the use of derivatives of naturally occurring L-amino acid derivatives. During the condensation process two diastereomers can be formed with the substituents in either a 1,3-*cis* or 1,3-*trans* arrangement. Since our reaction design of the above-mentioned reaction (depicted in Scheme 1b) showed a strong preference to use the 1,3-*cis*-disubstituted derivatives we concentrated our efforts on the isolation of these isomers.

Methyl amides **9** were produced from the corresponding methyl or ethyl esters via reaction with ethanolic methyl amide solution [26]. Solid methyl amides were further recrystallized to achieve a higher purity. Protected methyl amides **9a** and **9c** were synthesized in five or three literature-known steps from commercially available substrates, respectively [27–29].

The different methyl amides were subjected to the reaction with 1-naphthyl carbaldehyde employing 4 Å molecular sieves as dehydrating agent and 10 mol % Yb(OTf)_3_ in THF. This Lewis acid proved to be the Lewis acid of choice since the reaction proceeds without loss of optical purity [30]. The naphthyl residue was chosen since it revealed a high selectivity in the desired organocatalytic transformation. Table 1 depicts the scope with respect to 1-naphthyl carbaldehyde. Crude ^1^H NMR data of the reaction mixture showed in every case approximately 90% product formation of both diastereomers together, using an internal standard. The corresponding 1,3-*trans* diastereomers were not isolated in pure form. As reaction partners different methyl amides **9a–f** were employed in the reaction. The use of methyl-protected L-histidine-derived methyl amide **9a** (Table 1, entry 1) gave the desired imidazolidinone **7b** in 42% yield, whereas the unprotected derivative showed no conversion, probably because of its insolubility. The unprotected as well as the benzyl-protected L-tryptophan-derived methyl amides **9b** and **9c** were converted in good yields to the corresponding imidazolidinones (Table 1, entries 2 and 3). In the case of **7c** it is important to use not more than 1.0 equivalent of the aldehyde, otherwise a further reaction with the indole moiety is observed.

**Table 1:** Scope of the reaction using 1-naphthyl carbaldehyde (**10a**).^a^

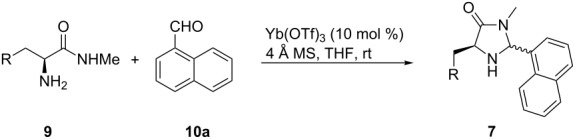					

Entry	Methyl amide **9**		Imidazolidinone **7**		Yield [%] (ratio)^b^

1	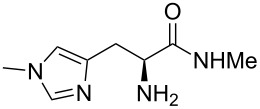	**9a**	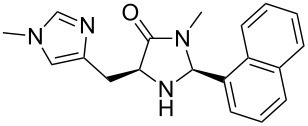	**7b**	42 (1:1)
2	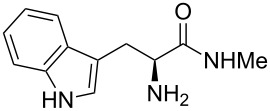	**9b**	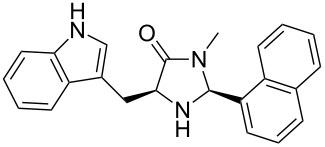	**7c**	45 (1:1)
3	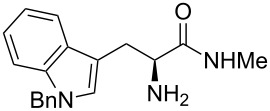	**9c**	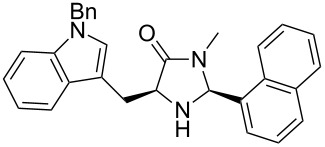	**7d**	42 (1:1)
4	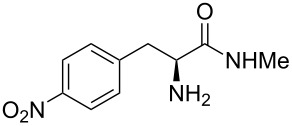	**9d**	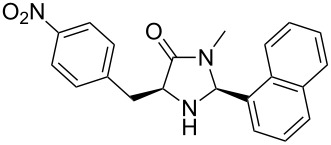	**7e**	12 (1:5)
5	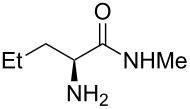	**9e**	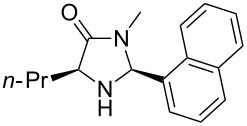	**7f**	46 (3:2)
6	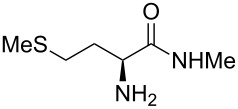	**9f**	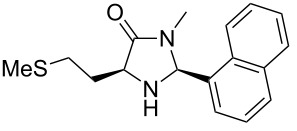	**7g**	32 (2:3)

^a^Reaction conditions: **9** (1.0 equiv), **10a** (0.9–1.1 equiv), Yb(OTf)_3_ (10 mol %), THF (4–12 mL/mmol), 4 Å MS (40 mg/mL), rt, 24 h. ^b^Isolated yield after column chromatography; the yield of both diastereomers together is in every case about 80–90%; the ratio of the two diastereomers is given in brackets, the desired *cis*-isomer is underlined (determined via ^1^H NMR spectroscopy of the crude reaction mixture).

A significant change in selectivity was observed while using electron-withdrawing methyl amide **9d**; the influence of the *para*-nitro substituent decreases the yield of the desired *cis*-diastereomer to only 12%. Utilization of non-aromatic methyl amides **9e** and **9f**, synthesized from L-norvaline and L-methionine methyl esters, gave rise to the desired imidazolidinones **7f** and **7g** (Table 1, entries 5 and 6) in 46% and 32%, respectively.

After evaluating the scope of imidazolidinones **7** we were keen to investigate which sterically more demanding aldehydes could be employed. Since the anticipated organocatalytic transformation (shown in Scheme 1b) delivered relatively high selectivity with imidazolidinones derived from L-phenylalanine this amino acid derivative was used as coupling partner. 1-Pyrene carbaldehyde **10b** gave only a minor excess of the *cis*-diastereomer (*S*,*S*)-**3b**. For mesityl carbaldehyde **10c** the desired isomer was obtained in 52% yield whereas the more sterically demanding aldehyde **10d** led to a switch in the selectivity, giving the desired (*S*,*S*)-diastereomer in only 23% yield (Table 2).

**Table 2:** Scope of the imidazolidinone formation with respect to aldehyde **10**.^a^

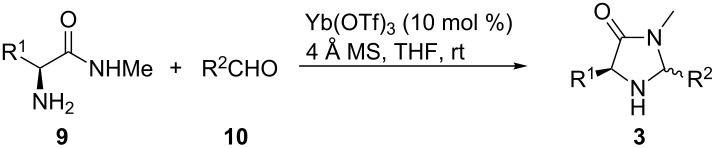						

Entry	Methyl amide **9**		Aldehyde **10**		Imidazolidinone **3**	Yield [%] (ratio)^b^

1	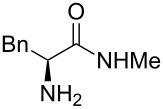	**9g**	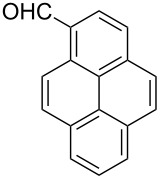	**10b**	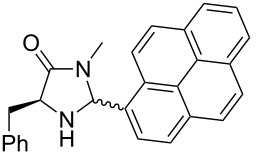	
						(*R*,*S*)-**3b**, 41
						(*S*,*S*)-**3b**, 49
2^c^	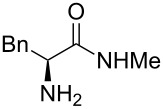	**9g**	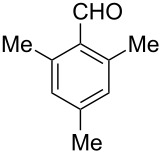	**10c**	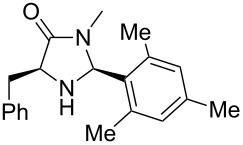	**3c**, 52 (3:2)
3^c^	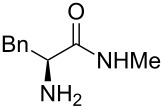	**9g**	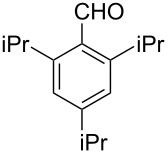	**10d**	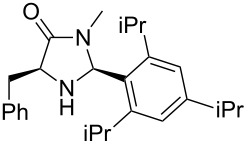	**3d**, 23 (1:2)

^a^Reaction conditions: **9** (1.0 equiv), **10** (1.1–1.5 equiv), Yb(OTf)_3_ (10 mol %), THF (4–5 mL/mmol), 4 Å MS (40 mg/mL), rt, 24 h. ^b^Isolated yield after column chromatography. The ratio of the two diastereomers is given in brackets, the desired *cis*-isomer is underlined (determined via ^1^H NMR spectroscopy of the crude reaction mixture). ^c^Combined yield of both diastereomers is 89% (entry 2) and 78% (entry 3), respectively.

## Conclusion

We were able to synthesize 10 different imidazolidone-based organocatalysts with yields of the desired 1,3-*cis*-disubstituted diastereomers of up to 52%. Different methyl amides derived from α-amino acids and bulky aromatic carbaldehydes were employed to access the MacMillan-type catalysts. The key to the success is the activation with ytterbium triflate as Lewis acid and the use of dehydrating molecular sieves. The prepared organocatalysts will be a useful contribution for the screening of a multitude of different organocatalytic transformations.

## Experimental

**General.** All solvents were distilled before use unless otherwise stated. Tetrahydrofuran (THF) was distilled over sodium and benzophenone under an argon atmosphere. Air- and moisture-sensitive reactions were carried out in oven-dried or flame-dried glassware, septum-capped under atmospheric pressure of argon. Commercially available compounds were used without further purification unless otherwise stated.

Proton (^1^H) and carbon (^13^C) NMR spectra were recorded on a 300, 400 or 600 MHz instrument using the residual signals from CHCl_3_, δ = 7.26 ppm and δ = 77.0 ppm, or MeOH, δ = 3.31 ppm and δ = 49.2 ppm, using TMS as internal reference for ^1^H and ^13^C chemical shifts, respectively. Assignments of the respective signals and the stereochemistry were made by combination of H,H-COSY, HSQC, HMBC and NOESY experiments. In some cases signals in the ^13^C NMR spectrum are missing because of bad relaxation of the respective signals. ESI high-resolution mass spectrometry was carried out on a FTICR instrument. IR spectra were measured on an ATR spectrometer. Optical rotation was measured on a common polarimeter.

**General procedure (GP) for the synthesis of imidazolidinones of type 3 and 7.** A Schlenk flask was charged with a magnetic stirring bar and powdered 4 Å molecular sieves (20 mg/mL) and flame-dried for 10 minutes under high vacuum. After cooling to ambient temperature methyl amide (1.0 equiv) was added and the flask subjected to the glovebox. After addition of Yb(OTf)_3_ (10 mol %) the flask was removed from the glovebox and THF (4.0 to 12.0 mL/mmol) and the corresponding aldehyde (0.9 to 1.2 equiv) were added subsequently. The resulting mixture was stirred at ambient temperature for 24 h. The suspension was filtered over Celite and the solvent was removed in vacuo.

**(2*S*,5*S*)-5-((*N*-Methyl-1*H*-imidazol-4-yl)methyl)-3-methyl-2-(naphthalen-1-yl)imidazolidin-4-one (7b).** (*S*)-*N*-Methylhistidine methyl amide (190 mg, 1.04 mmol, 1.0 equiv), 1-naphthyl carbaldehyde (180 mg, 156 µL, 1.15 mmol, 1.1 equiv), 4 Å molecular sieves (240 mg) and Yb(OTf)_3_ (64.5 mg, 104 µmol, 10 mol %) in THF (12 mL) were reacted according to the GP. Silica gel column chromatography (DCM/MeOH 1:0 → 50:1) afforded the desired (*S,S*)-diastereomer **7b** (140 mg, 437 µmol, 42%) as pale yellow oil: ^1^H NMR (200 MHz, MeOD) δ 2.65 (s, 3H), 3.05 (dd, *J* = 9.4, 5.1 Hz, 2H), 3.70 (s, 3H), 3.91 (t, *J* = 5.1 Hz, 1H), 6.09 (br s, 1H), 6.87–6.98 (m, 1H), 6.97–7.12 (m, 1H), 7.43–7.58 (m, 4H), 7.84–8.00 (m, 2H), 8.02–8.16 (m, 1H); ^13^C NMR (150 MHz, CDCl_3_) δ 28.7, 33.3, 60.4, 60.4, 118.3, 122.7, 125.1, 125.1, 126.0, 126.6, 126.7, 128.8, 137.2, 137.3, 137.8 (the carbonyl signal as well as one aliphatic, one aromatic CH and one aromatic C_q_-signal are missing); 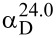 (*c* 1.0, CHCl_3_) +38.0°; IR (ATR) 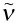 (cm^−1^): 3349, 2922, 1683, 1261, 1159; HRMS (ESI) *m/z*: calcd for C_19_H_20_N_4_O, 343.1529; found, 343.1529.

**(2*S*,5*S*)-5-((Indol-3-yl)methyl)-3-methyl-2-(naphthalen-1-yl)imidazolidin-4-one (7c).** (*S*)-Tryptophan methyl amide (340 mg, 1.56 mmol, 1.0 equiv), 1-naphthyl carbaldehyde (243 mg, 212 µL, 1.56 mmol, 1.0 equiv), 4 Å molecular sieves (160 mg) and Yb(OTf)_3_ (97.0 mg, 156 µmol, 10 mol %) in THF (8.0 mL) were reacted according to the GP. Silica gel column chromatography (*n*-pentane/EtOAc 1:1 → 2:3 → 0:1) gave the desired (*S,S*)-diastereomer **7c** (250 mg, 203 µmol, 45%) as pale yellow solid: mp 145 °C; ^1^H NMR (300 MHz, MeOD) δ 2.52 (s, 3H), 3.23 (dd, *J* = 14.8, 5.3 Hz, 1H), 3.49 (dd, *J* = 14.8, 3.9 Hz, 1H), 3.85–3.96 (m, 1H), 5.98 (br s, 1H), 6.86–6.98 (m, 1H), 6.99–7.17 (m, 4H), 7.33–7.47 (m, 3H), 7.53 (d, *J* = 7.9 Hz, 1H), 7.69–7.87 (m, 3H); ^13^C NMR (75 MHz, MeOD) δ 30.6, 30.6, 64.3, 112.2, 115.0, 122.3, 122.7, 125.3, 125.6, 128.2, 128.9, 129.6, 130.3, 131.6, 132.5, 133.3, 133.4, 134.8, 137.9, 140.9, 180.3 (one aliphatic CH and one aromatic CH are missing); 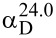 (*c* 1.0, CHCl_3_) −6.0°; IR (ATR) 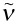 (cm^−1^): 3404, 3280, 1681, 1435, 1097; HRMS (ESI) *m/z*: calcd for C_23_H_21_N_3_O, 378.1577; found, 378.1578.

**(2*S*,5*S*)-5-((*N*-Benzylindol-3-yl)methyl)-3-methyl-2-(naphthalen1-yl)imidazolidin-4-one (7d).** (*S*)-*N*-Benzyltryptophan methyl amide (307 mg, 1.00 mmol, 1.0 equiv), 1-naphthyl carbaldehyde (156 mg, 136 µL, 1.10 mmol, 1.1 equiv), 4 Å molecular sieves (120 mg) and Yb(OTf)_3_ (62.0 mg, 100 µmol, 10 mol %) in THF (6.0 mL) were reacted according to the GP. Silica gel column chromatography (*n*-pentane/EtOAc 1:0 → 50:1) the desired (*S,S*)-diastereomer **7d** (188 mg, 422 µmol, 42%) as pale yellow solid: mp 120 °C (dec); ^1^H NMR (200 MHz, MeOD) δ 2.54 (s, 3H), 3.11–3.32 (dd, *J* = 14.9, 5.5 Hz, 1H), 3.51 (dd, *J* = 14.9, 3.6 Hz, 1H), 3.88–3.96 (m, 1H), 5.23 (s, 2H), 6.01 (br s, 1H), 6.87–7.22 (m, 11H), 7.32–7.48 (m, 3H), 7.55 (d, *J* = 7.9 Hz, 1H), 7.71 (d, *J* = 8.3 Hz, 1H), 7.83 (d, *J* = 8.4 Hz, 1H); ^13^C NMR (150 MHz, CDCl_3_) δ 26.3, 27.9, 49.9, 60.3, 109.3, 109.6, 119.6, 122.0, 122.3, 125.3, 126.0, 126.7, 126.7, 127.5, 127.8, 128.3, 128.6, 128.8, 131.0, 133.7, 136.8, 137.2, 176.1 (one aliphatic, two aromatic CH and one aromatic C_q_-signals are missing); 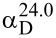 (*c* 1.0, CHCl_3_) −6.0°; IR (ATR) 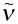 (cm^−1^): 3491, 1684, 1259, 1170, 1035; HRMS (ESI) *m/z*: calcd for C_30_H_27_N_3_O, 468.2046; found, 468.2050.

**(2*S*,5*S*)-5-(*p*-Nitrobenzyl)-3-methyl-2-(naphthalen-1-yl)imidazolidin-4-one (7e).** (*S*)-*p*-Nitrophenylalanine methyl amide (892 mg, 4.00 mmol, 1.0 equiv), 1-naphthyl carbaldehyde (625 mg, 543 µL, 4.00 mmol, 1.0 equiv), 4 Å molecular sieves (480 mg) and Yb(OTf)_3_ (248 mg, 400 µmol, 10 mol %) in THF (24 mL) were reacted according to the GP. Silica gel column chromatography (*n*-pentane/EtOAc 2:1 → 1:1 → 0:1) gave the desired (*S,S*)-diastereomer **7e** (180 mg, 498 µmol, 12%) as pale yellow solid: mp 75 °C; ^1^H NMR (300 MHz, CDCl_3_) δ 2.71 (s, 3H), 3.16–3.37 (m, 2H), 4.02 (dd, *J* = 5.1, 5.1 Hz, 1H), 5.99 (br s, 1H), 7.02 (br s, 1H), 7.28–7.41 (m, 3H), 7.41– 7.57 (m, 2H), 7.79–7.94 (m, 2H), 7.94–8.06 (m, 1H), 8.05–8.13 (m, 2H); ^13^C NMR (75 MHz, CDCl_3_) δ 28.0, 37.3, 60.4, 77.2, 122.3, 123.7, 125.2, 126.2, 126.8, 129.1, 130.0, 130.6, 131.0, 134.0, 144.9, 147.0 (the carbonyl and two aromatic CH signals are missing); 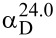 (*c* 1.0, CHCl_3_) −26.0°; IR (ATR) 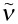 (cm^−1^): 3324, 1690, 1513, 1342, 1107; HRMS (ESI) *m/z*: calcd for C_21_H_19_N_3_O_3_, 384.1319; found, 384.1319.

**(2*S*,5*S*)-3-Methyl-2-(naphthalen-1-yl)-5-propylimidazolidin-4-one (7f).** (*S*)-Norvaline methyl amide (521 mg, 4.00 µmol, 1.0 equiv), 1-naphthyl carbaldehyde (562 mg, 489 µL, 3.60 mmol, 0.9 equiv), 4 Å molecular sieves (480 mg) and Yb(OTf)_3_ (248 mg, 400 µmol, 10 mol %) in THF (24 mL) were reacted according to GP. Silica gel column chromatography (*n*-pentane/EtOAc 4:1 → 1:1) gave the desired (*S,S*)-diastereomer **7f** (440 mg, 1.64 mmol, 46%) as pale yellow oil. ^1^H NMR (200 MHz, CDCl_3_) δ 0.94 (t, *J* = 7.2 Hz, 3H), 1.34–1.71 (m, 3H), 1.87 (br s, 1H, N*H*), 1.89–2.13 (m, 1H), 3.57–3.70 (m, 1H), 6.01 (br s, 1H), 7.40–7.61 (m, 4H), 7.83–8.00 (m, 2H), 8.15–8.29 (m, 1H); ^13^C NMR (75 MHz, CDCl_3_) δ 13.9, 19.2, 28.1, 34.4, 59.8, 77.2, 122.6, 125.4, 126.2, 126.9, 129.0, 129.8, 131.2, 131.4, 133.7, 134.1, 176.5; 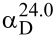 (*c* 1.0, CHCl_3_) +89.0°; IR (ATR) 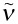 (cm^−1^): 3321, 2957, 1689, 1396, 1322; HRMS (ESI) *m/z*: calcd for C_17_H_20_N_2_O, 291.1468; found, 291.1468.

**(2*S*,5*S*)-3-Methyl-5-(2-(methylthio)ethyl)-2-(naphthalen-1-yl)imidazolidin-4-one (7g).** (*S*)-Methionine methyl amide (649 mg, 4.00 µmol, 1.0 equiv), 1-naphthyl carbaldehyde (562 mg, 489 µL, 3.60 mmol, 0.9 equiv), 4 Å molecular sieves (480 mg) and Yb(OTf)_3_ (248 mg, 400 µmol, 10 mol %) in THF (24 mL) were reacted according to the GP. Silica gel column chromatography (*n*-pentane/EtOAc 2:1 → 1:2) gave the desired (*S,S*)-diastereomer **7g** (350 mg, 1.17 mmol, 32%) as yellow oil. ^1^H NMR (300 MHz, CDCl_3_) δ 1.83–1.98 (m, 1H), 2.07 (s, 3H), 2.19 (br s, 1H, N*H*), 2.24–2.38 (m, 1H), 2.58–2.72 (m, 2H), 2.75 (s, 3H), 3.75–3.89 (m, 1H), 6.03 (br s, 1H), 7.45–7.60 (m, 4H), 7.85–7.97 (m, 3H), 8.20–8.25 (m, 1H); ^13^C NMR (75 MHz, CDCl_3_) δ 15.2, 28.0, 30.6, 31.5, 58.7, 77.2, 122.6, 125.4, 126.2, 126.9, 129.0, 129.9, 131.2, 133.6, 134.1, 175.5 (one aromatic CH-signal is missing); 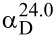 (*c* 1.0, CHCl_3_) +66.0°; IR (ATR) 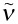 (cm^−1^): 3321, 2957, 1689, 1396, 1322; HRMS (ESI) *m/z*: calcd for C_17_H_20_N_2_OS, 323.1189; found, 323.1191.

**(2*R*,5*S*)-5-Benzyl-3-methyl-2-(pyren-1-yl)imidazolidin-4-one ((*R*,*S*)-3b) and (2*S*,5*S*)-5-benzyl-3-methyl-2-(pyren-1-yl)imidazolidin-4-one ((*S*,*S*)-3b).** (*S*)-Phenylalanine methyl amide (0.89 g, 5.0 mmol, 1.0 equiv), pyrene-1-carbaldehyde (1.73g, 7.50 mmol, 1.5 equiv), 4 Å molecular sieves (400 mg) and Yb(OTf)_3_ (310 mg, 500 µmol, 10 mol %) in THF (20 mL) were reacted according to the GP. Silica gel column chromatography (*n*-pentane/EtOAc 4:1 → 2:1 → 1:2) gave the faster eluting (*R,S*)-diastereomer (*R*,*S*)-**3b** (801 mg, 2.05 mmol, 41%) and the desired (*S,S*)-diastereomer (*S*,*S*)-**3b** (952 mg, 2.44 mmol, 49%) as yellow solids. Analytical data of (*R*,*S*)-**3b**: mp 70 °C; ^1^H NMR (600 MHz, CDCl_3_) δ 2.36 (br s, 1H), 2.70 (s, 3H), 3.10–3.24 (m, 2H), 4.15 (dd, *J* = 5.2, 5.2 Hz, 1H), 5.74 (s, 1H), 7.29 (ddt, *J* = 9.2, 7.4, 1.2 Hz, 1H), 7.34–7.39 (m, 2H), 7.41–7.45 (m, 2H), 7.82 (d, *J* = 7.4 Hz, 1H), 8.02–8.07 (m, 2H), 8.10 (d, *J* = 9.2 Hz, 1H), 8.15–8.24 (m, 5H); ^13^C NMR (150 MHz, CDCl_3_) δ 27.9, 38.8, 60.2, 121.6, 122.8, 124.6, 125.1, 125.2, 125.5, 125.8, 126.3, 126.8, 127.2, 128.1, 128.4, 128.6, 129.1, 129.9, 130.5, 131.2, 131.2, 131.7, 137.7, 175.4 (one aliphatic CH-signal is missing); 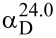 (*c* 1.0, CHCl_3_) −62.0°; IR (ATR) 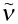 (cm^−1^): 3310, 3029, 1688, 1395, 1086; HRMS (ESI) *m/z*: calcd for C_27_H_22_N_2_O, 413.1624; found, 413.1626. Analytical data of (*S*,*S*)-**3b**: mp 95 °C; ^1^H NMR (600 MHz, CDCl_3_) δ 2.68 (s, 3H), 3.18 (br d, *J* = 14.2 Hz, 1H), 3.36 (dd, *J* = 14.2, 5.4 Hz, 1H), 4.04 (t, *J* = 5.4 Hz, 1H), 6.32 (br s, 1H), 7.17–7.25 (m, 2H), 7.24–7.31 (m, 5H), 7.95–8.01 (m, 1H), 8.01–8.08 (m, 2H), 8.11 (d, *J* = 8.9 Hz, 1H), 8.19–8.25 (m, 2H), 8.28–8.39 (m, 1H); ^13^C NMR (75 MHz, CDCl_3_) δ 27.9, 36.7, 60.9, 121.3, 124.6, 124.8, 125.3, 125.5, 125.8, 126.2, 127.0, 127.2, 128.2, 128.6, 128.9, 129.4, 129.8, 130.5, 131.2, 136.5, 175.0 (one aliphatic, two aromatic CH-signals as well as one aromatic C_q_-signal are missing); 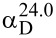 (*c* 1.0, CHCl_3_) −10.0°; IR (ATR) 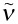 (cm^−1^): 3475, 2914, 1682, 1255, 1033; HRMS (ESI) *m/z*: calcd for C_27_H_22_N_2_O, 413.1624; found, 413.1627.

**(2*S*,5*S*)-5-Benzyl-3-methyl-2-mesitylimidazolidin-4-one (3c).** (*S*)-Phenylalanine methyl amide (71.3 mg, 400 µmol, 1.0 equiv), mesityl carbaldehyde (65.2 mg, 64.9 µL, 440 µmol, 1.1 equiv), 4 Å molecular sieves (40 mg) and Yb(OTf)_3_ (25 mg, 40 µmol, 10 mol %) in THF (2.0 mL) were reacted according to the GP. Silica gel column chromatography (*n*-pentane/EtOAc 3:1 → 1:1) gave the desired (*S,S*)-diastereomer **3c** (64.1 mg, 208 µmol, 52%) as a white solid: mp 75 °C; ^1^H NMR (600 MHz, CDCl_3_) δ 1.80 (s, 3H), 2.24 (s, 3H), 2.37 (s, 3H), 2.58 (s, 3H), 3.18 (ddd, *J* = 14.0, 6.9, 0.9 Hz, 1H), 3.24 (ddd, *J* = 14.0, 3.8, 0.9 Hz, 1H), 3.83 (ddd, *J* = 6.9, 3.8, 1.7 Hz, 1H), 5.72 (d, *J* = 1.7 Hz, 1H), 6.75 (s, 1H), 6.83 (s, 1H), 7.20–7.24 (m, 1H), 7.25–7.28 (m, 4H); ^13^C NMR (150 MHz, CDCl_3_) δ 18.4, 20.3, 20.6, 26.8, 35.8, 60.6, 72.8, 126.7, 128.2, 128.5, 129.5, 129.6, 131.5, 137.1, 137.4, 137.9, 138.4, 173.8; 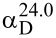 (*c* 1.0, CHCl_3_) −62.0°; IR (ATR) 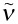 (cm^−1^): 3315, 2921, 1693, 1433, 1309; HRMS (ESI) *m/z*: calcd for C_20_H_24_N_2_O, 331.1781; found, 331.1781.

**(2*S*,5*S*)-5-Benzyl-3-methyl-2-(2,4,6-triisopropylphen-1-yl)imidazolidin-4-one (3d).** (*S*)-Phenylalanine methyl amide (217 mg, 1.00 µmol, 1.0 equiv), 2,4,6-triisopropylbenzaldehyde (255 mg, 273 µL, 1.10 mmol, 1.1 equiv), 4 Å molecular sieves (80 mg) and Yb(OTf)_3_ (62.1 mg, 100 µmol, 10 mol %) in THF (4.0 mL) were reacted according to the GP. Silica gel column chromatography (*n*-pentane/EtOAc 10:1 → 2:1) gave the desired (*S,S*)-diastereomer **3d** (90.2 mg, 230 µmol, 23%) as a colorless oil: ^1^H NMR (600 MHz, CDCl_3_) δ 1.13 (d, *J* = 6.9 Hz, 3H), 1.15 (d, *J* = 6.9 Hz, 3H), 1.24 (d, *J* = 6.9 Hz, 12H), 2.69 (s, 3H), 2.83–2.90 (m, 2H), 3.30 (hept, *J* = 6.9 Hz, 1H), 3.41 (dd, *J* = 14.1, 3.4 Hz, 1H), 3.46 (hept, *J* = 6.9 Hz, 1H), 3.84 (d, *J* = 8.7 Hz, 1H), 5.92 (br s, 1H), 7.00 (d, *J* = 1.9 Hz, 1H), 7.07 (d, *J* = 1.9 Hz, 1H), 7.17–7.25 (m, 1H), 7.26–7.30 (m, 4H); ^13^C NMR (150 MHz, CDCl_3_) δ 23.8, 23.8, 24.2, 24.3, 24.7, 25.7, 27.5, 27.5, 29.1, 34.1, 37.7, 61.3, 71.5, 121.2, 123.6, 126.0, 126.6, 128.6, 129.2, 138.2, 148.4, 149.7, 150.1, 173.7; 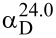 (*c* 1.0, CHCl_3_) −102°; IR (ATR) 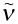 (cm^−1^): 3548, 2957, 1683, 1397, 1098; HRMS (ESI) *m/z*: calcd for C_26_H_36_N_2_O, 415.2720; found, 415.2722.

## Supporting Information

File 1 Copies of ^1^H and ^13^C NMR spectra.
