# Supplementary material for: Synthesis of 1,3-cis-disubstituted sterically encumbered imidazolidinone organocatalysts
Source: Beilstein J Org Chem. 2017 Dec 1;13:2577–83. doi: 10.3762/bjoc.13.254 (PMC5727825; doi:10.3762/bjoc.13.254)
Supplement: File 1 — Copies of 1H and 13C NMR spectra. [file Beilstein_J_Org_Chem-13-2577-s001.pdf]

Supporting Information

for

Synthesis of 1,3-*cis*-disubstituted sterically encumbered imidazolidinone organocatalysts

Jan Wallbaum and Daniel B. Werz*

Address: Institut für Organische Chemie, Technische Universität Braunschweig, Hagenring 30, 38106 Braunschweig, Germany

Email: Daniel B. Werz - d.werz@tu-braunschweig.de

* Corresponding author

Copies of ^{1}H and ^{13}C NMR spectra

(2*S*,5*S*)-(+)-5-((*N*-methylimidazol-4-yl)methyl)-3-methyl-2-(naphthalen-1-yl)imidazolidin-4-one (7b)

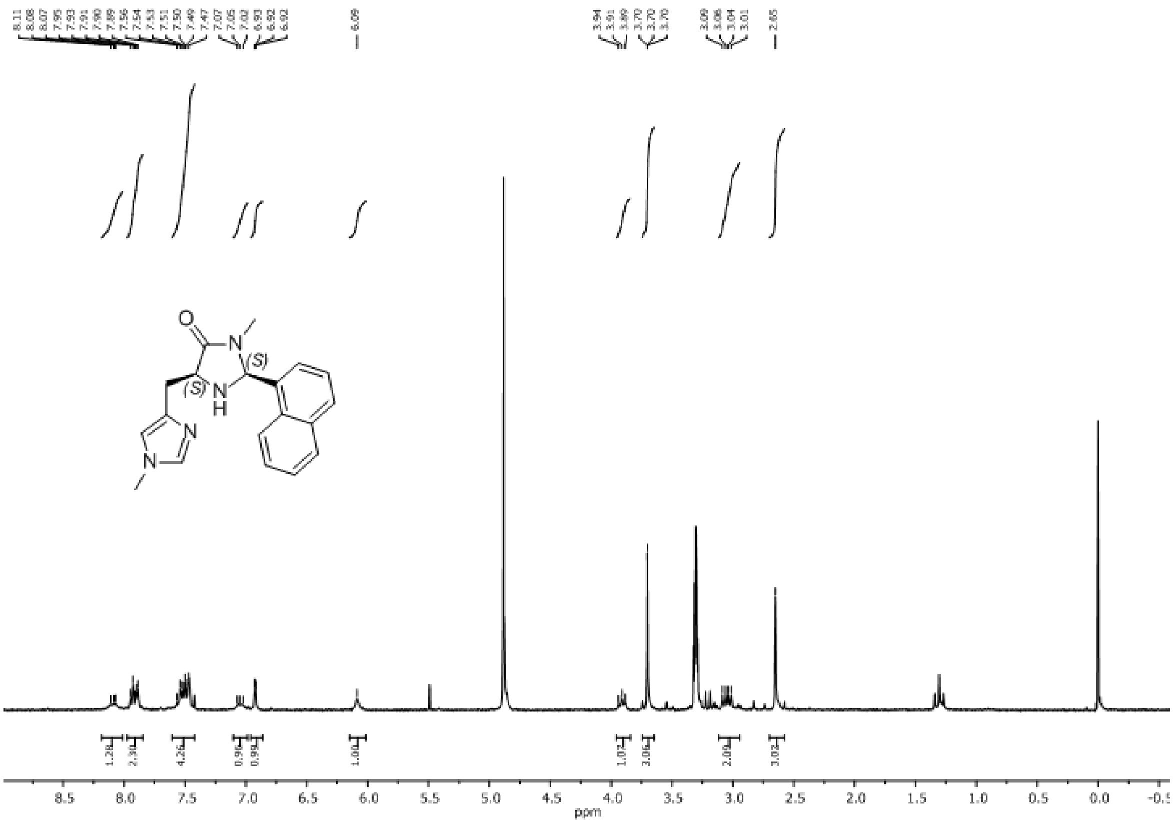

(^{1}H NMR, 200 MHz, MeOD)

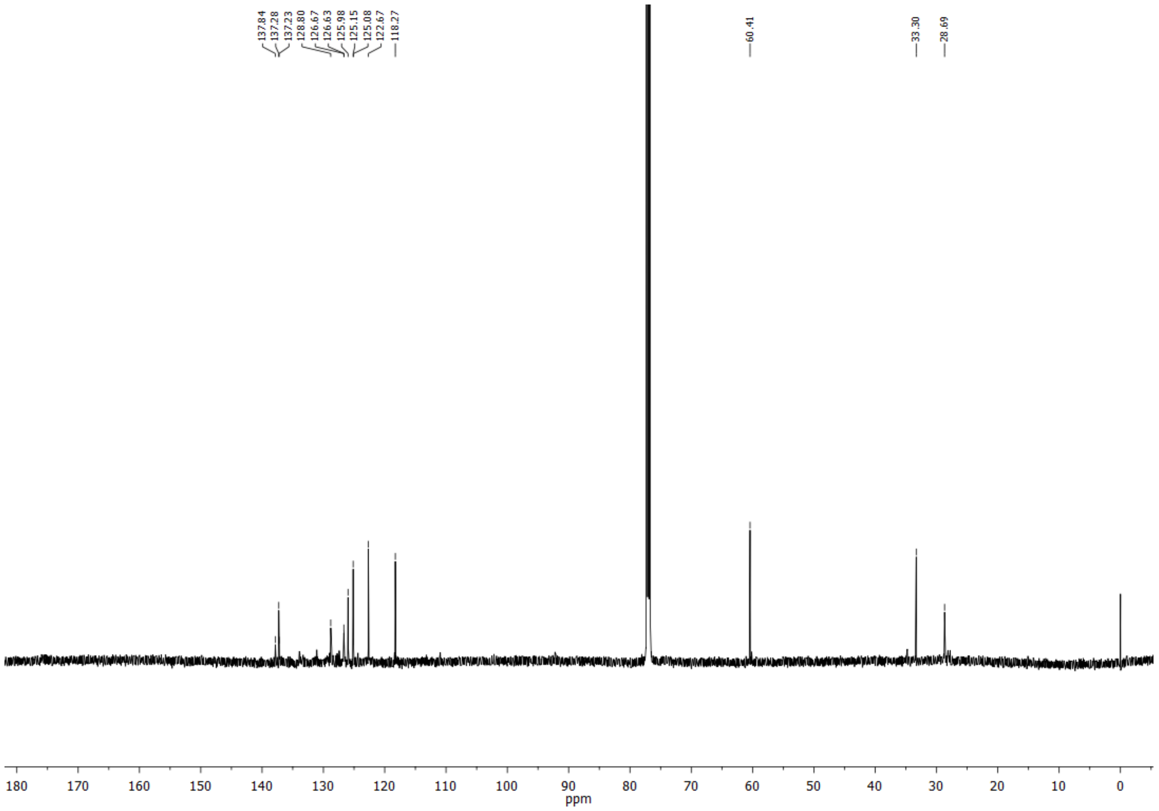

(^{13}C NMR, 150 MHz, $CDCl_3$)

(2*S*,5*S*)-(−)-5-((Indol-3-yl)methyl)-3-methyl-2-(naphthalen-1-yl)imidazolidin-4-one (7c)

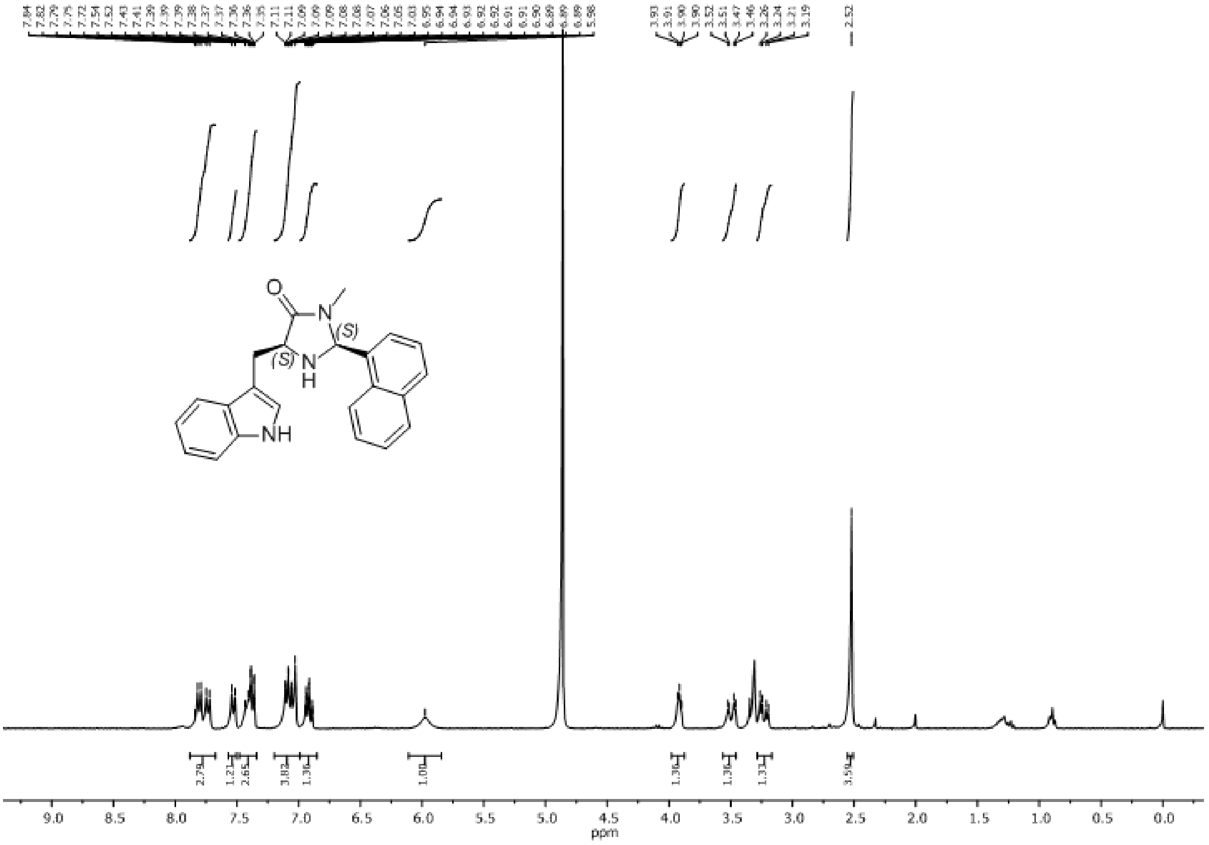

(^{1}H NMR, 300 MHz, MeOD)

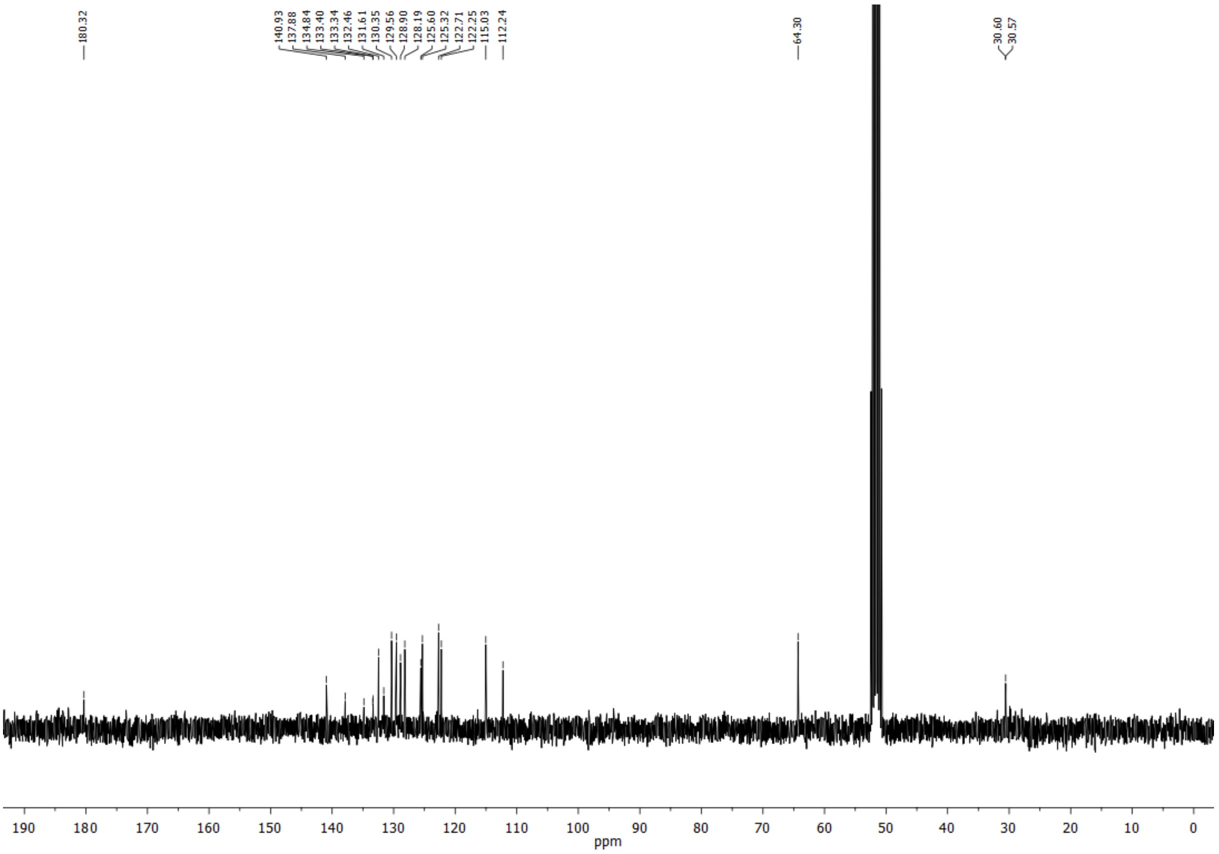

(^{13}C NMR, 75 MHz, MeOD)

(2*S*,5*S*)-(−)-5-((*N*-benzylindol-3-yl)methyl)-3-methyl-2-(naphthalen-1-yl)imidazolidin-4-one (7d)

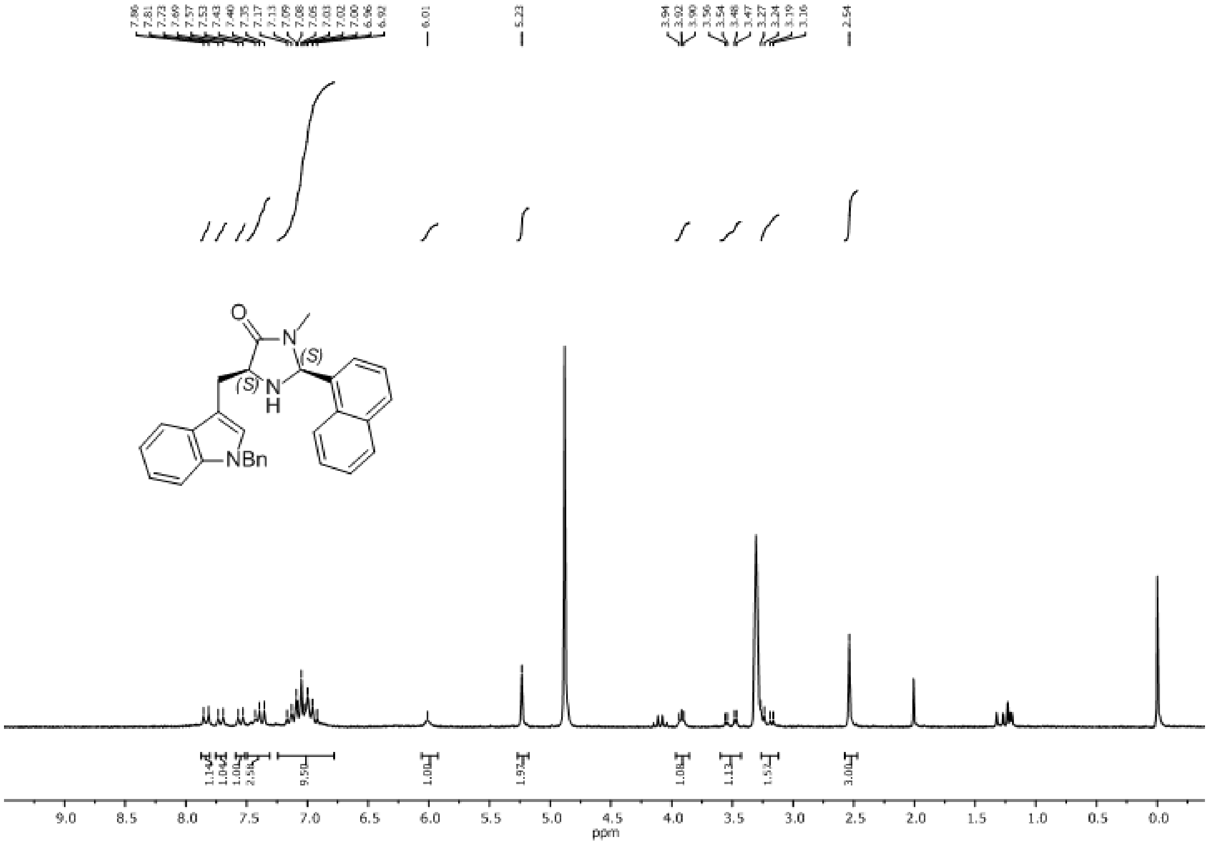

(^{1}H NMR, 200 MHz, MeOD)

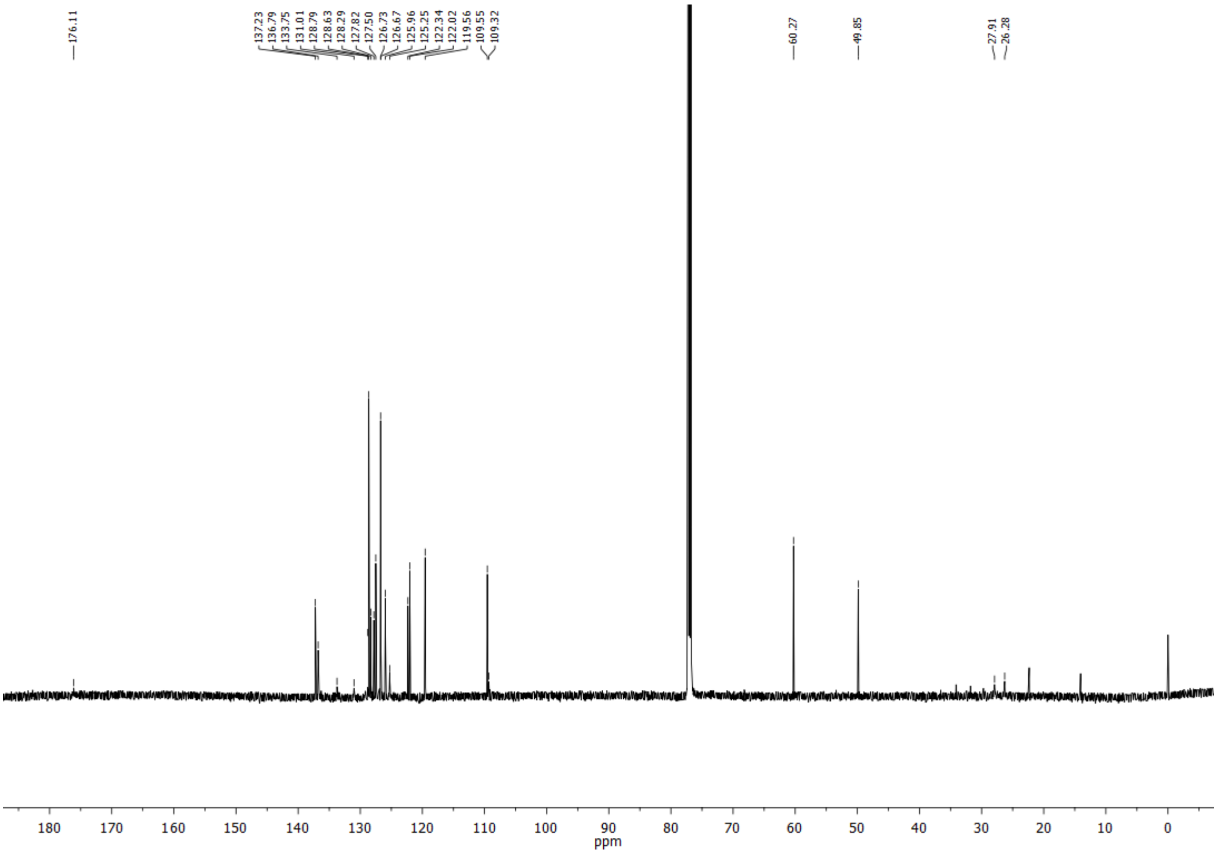

(^{13}C NMR, 150 MHz, $CDCl_3$)

(2*S*,5*S*)-(−)-5-(*p*-Nitrobenzyl)-3-methyl-2-(naphthalen-1-yl)imidazolidin-4-one (7e)

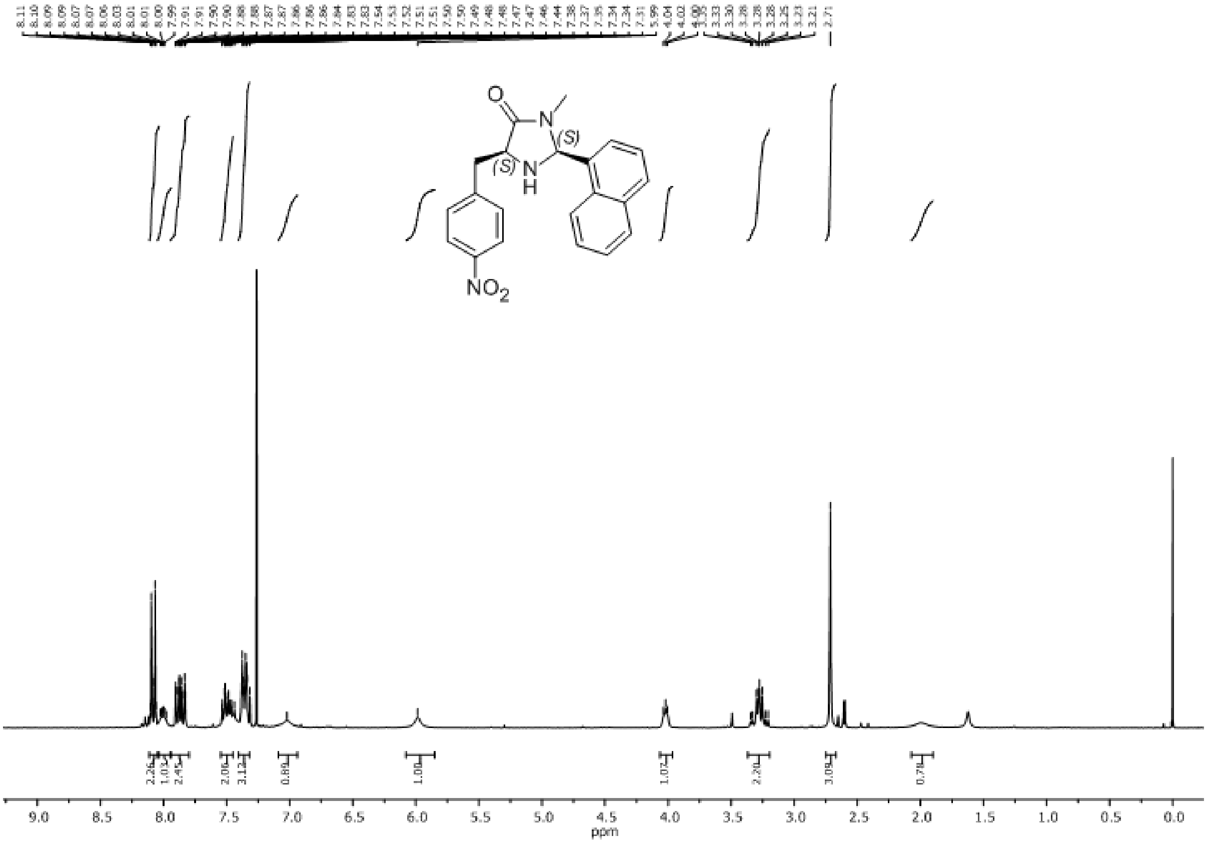

(^{1}H NMR, 300 MHz, $CDCl_3$)

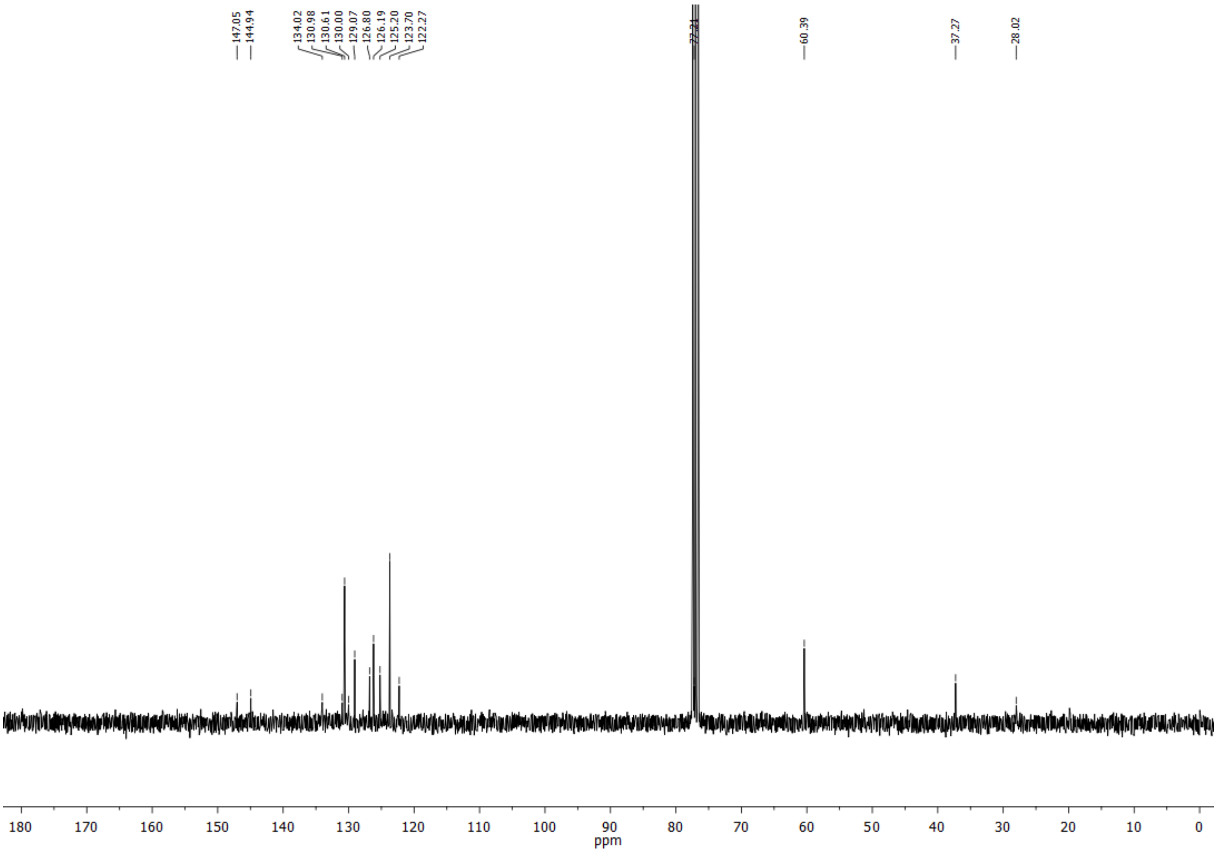

(^{13}C NMR, 75 MHz, $CDCl_3$)

(2*S*,5*S*)-3-Methyl-2-(naphthalen-1-yl)-5-propylimidazolidin-4-one (7f)

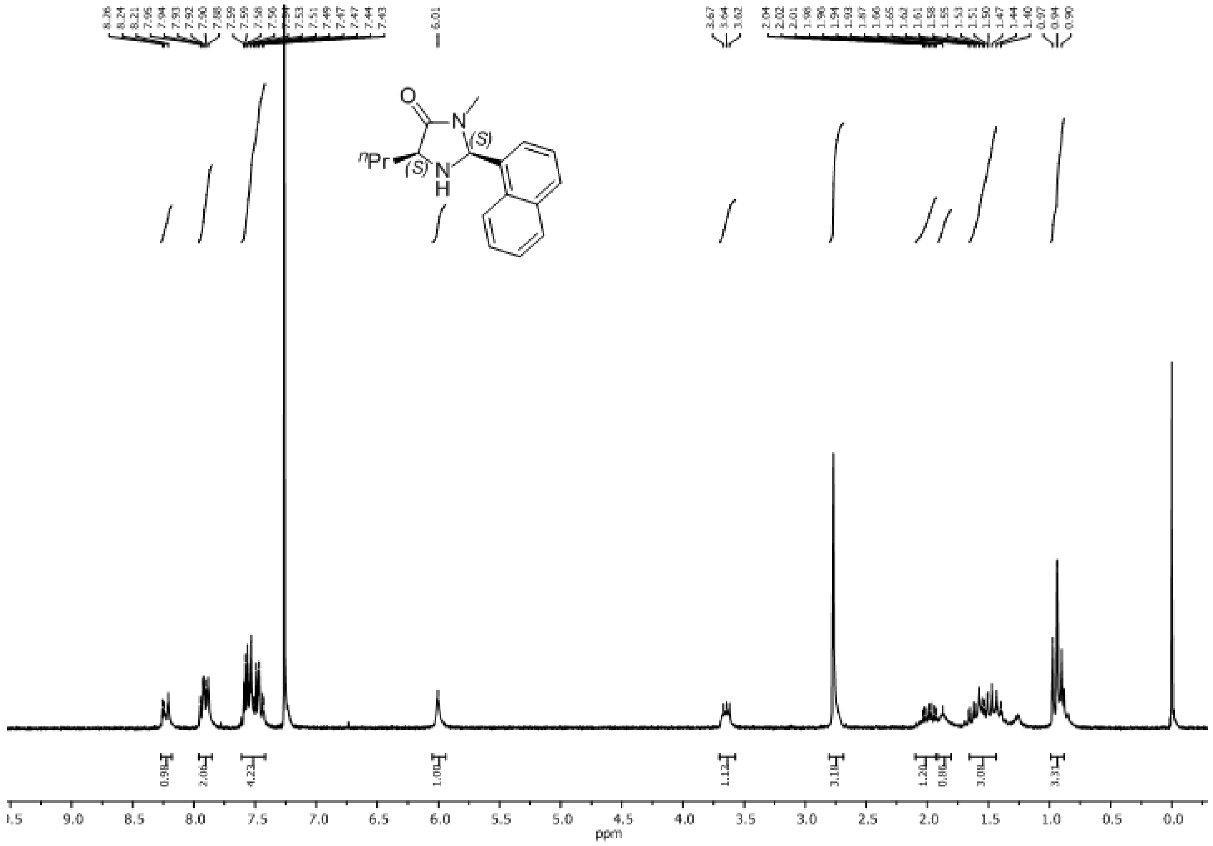

(^{1}H NMR, 200 MHz, $CDCl_3$)

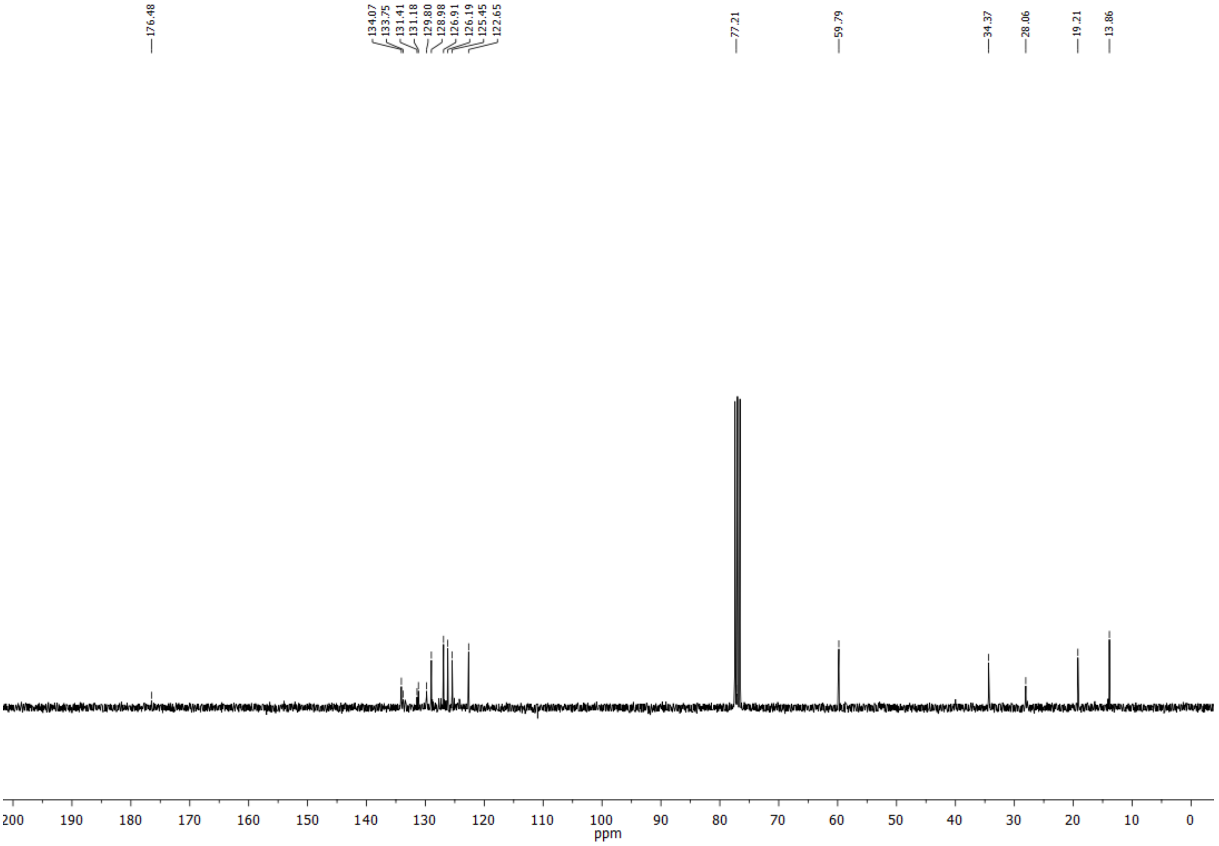

(^{13}C NMR, 75 MHz, $CDCl_3$)

(2S,5S)-3-Methyl-5-(2-(methylthio)ethyl)-2-(naphthalen-1-yl)imidazolidin-4-one (7g)

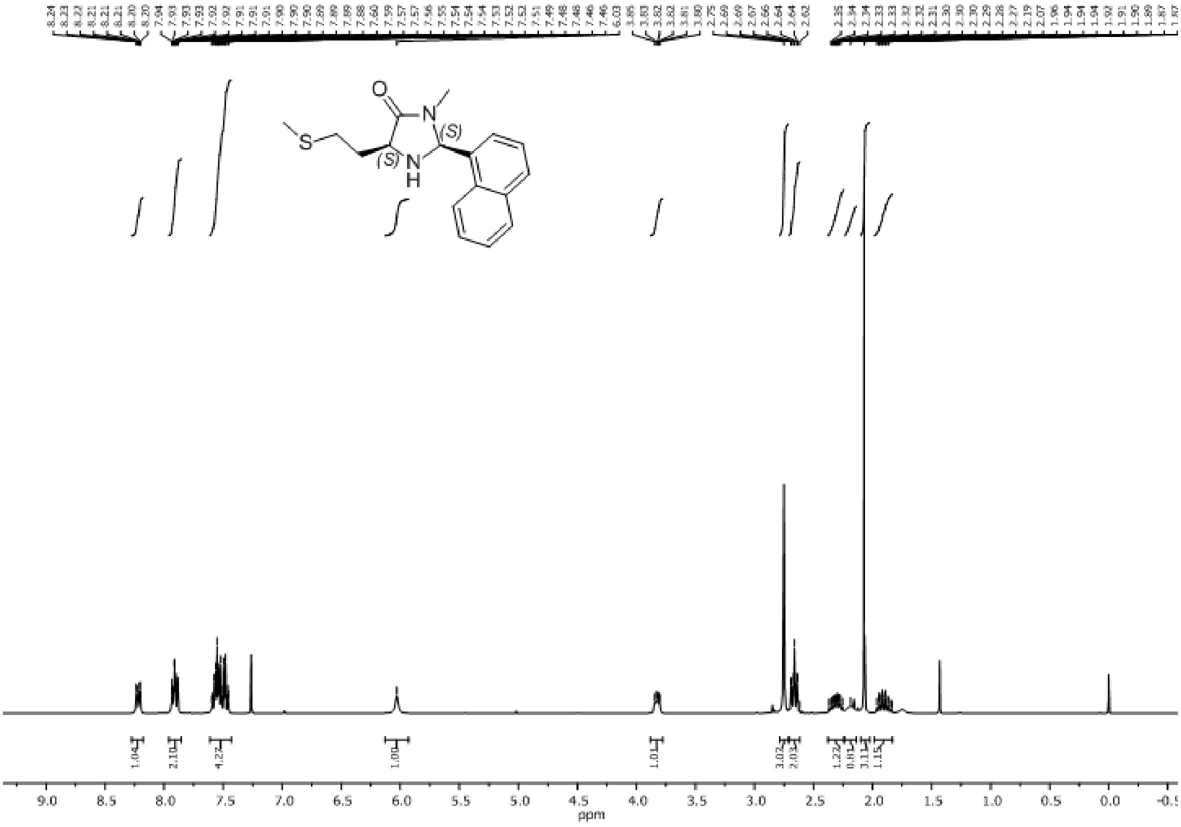

(^{1}H NMR, 300 MHz, $CDCl_3$)

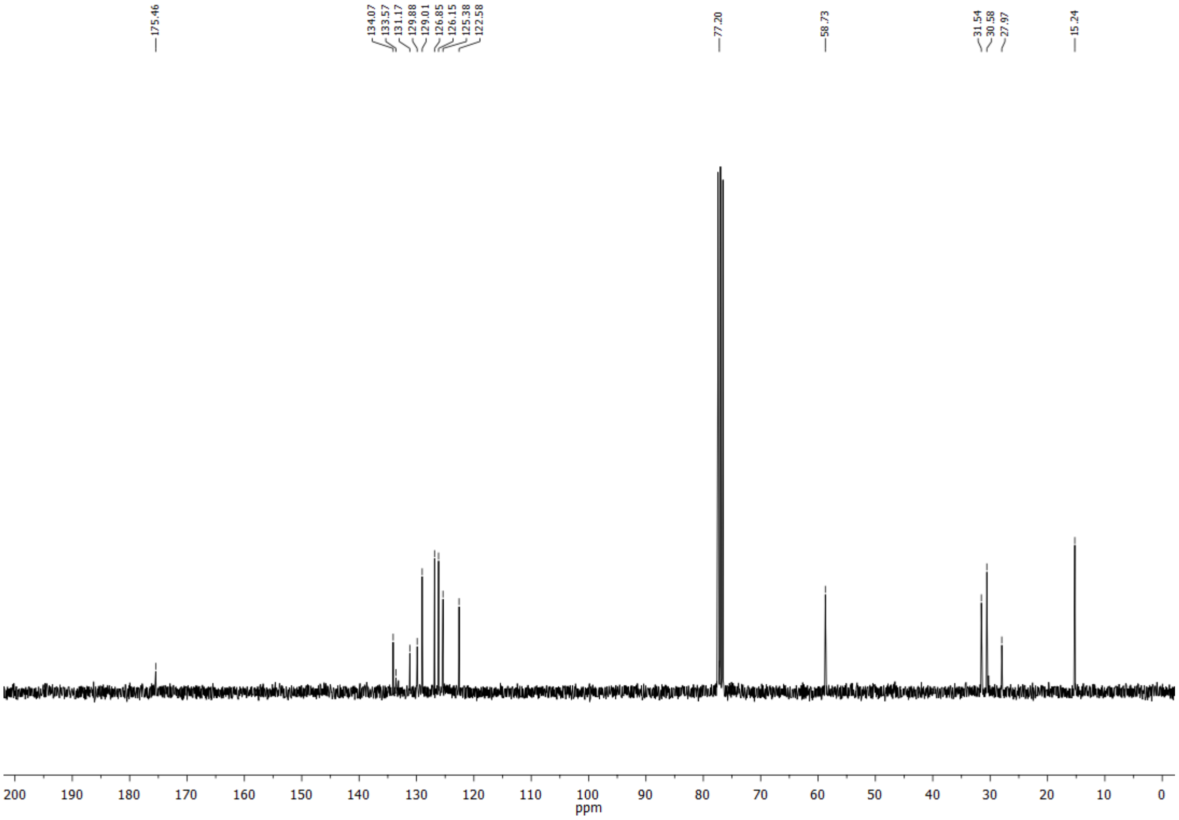

(^{13}C NMR, 75 MHz, $CDCl_3$)

(2*R*,5*S*)-(−)-5-Benzyl-3-methyl-2-(pyren-1-yl)imidazolidin-4-one ((*R*,5)-3b)

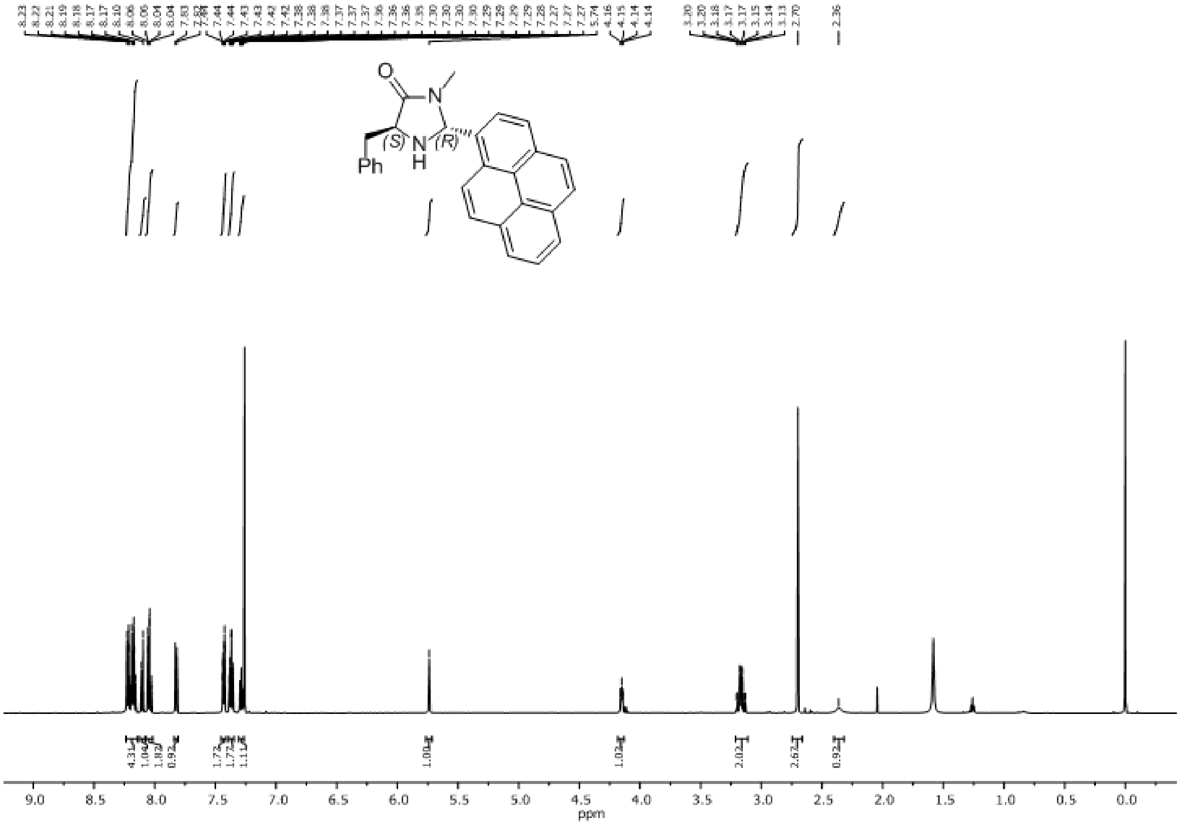

(^{1}H NMR, 600 MHz, $CDCl_3$)

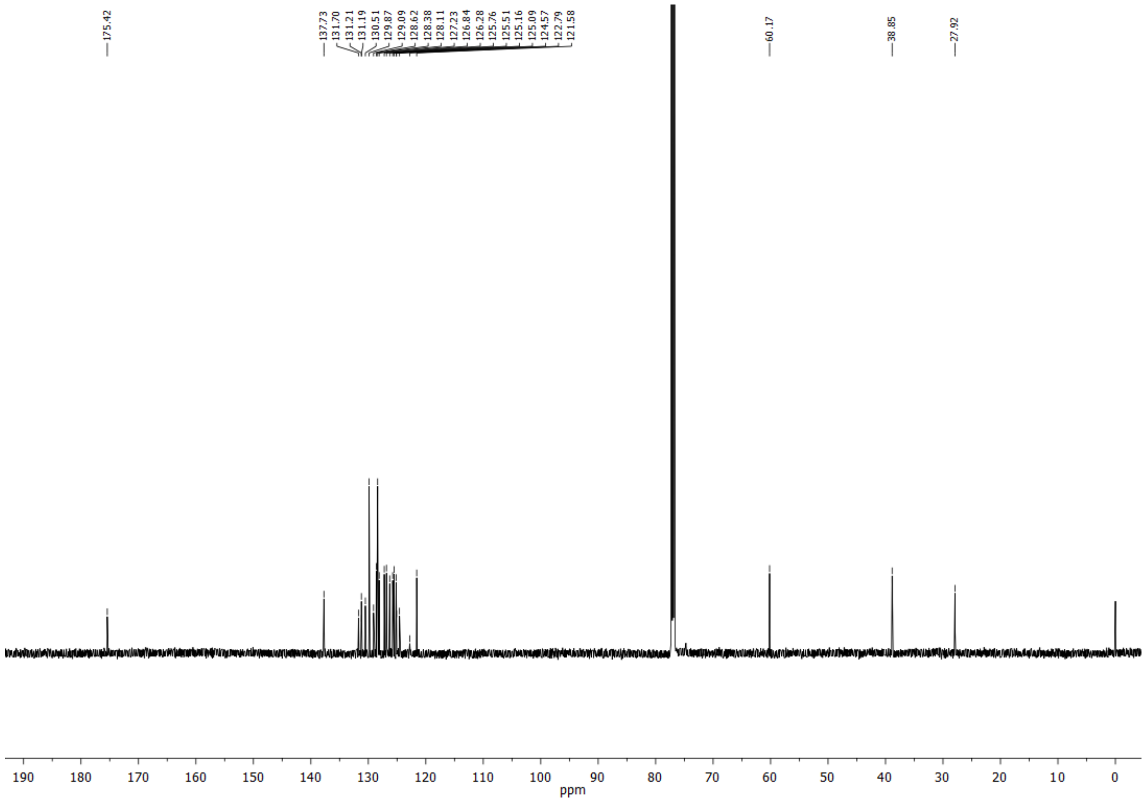

(^{13}C NMR, 150 MHz, $CDCl_3$)

(2*S*,5*S*)-(−)-5-Benzyl-3-methyl-2-(pyren-1-yl)imidazolidin-4-one ((*S*,5)-3b)

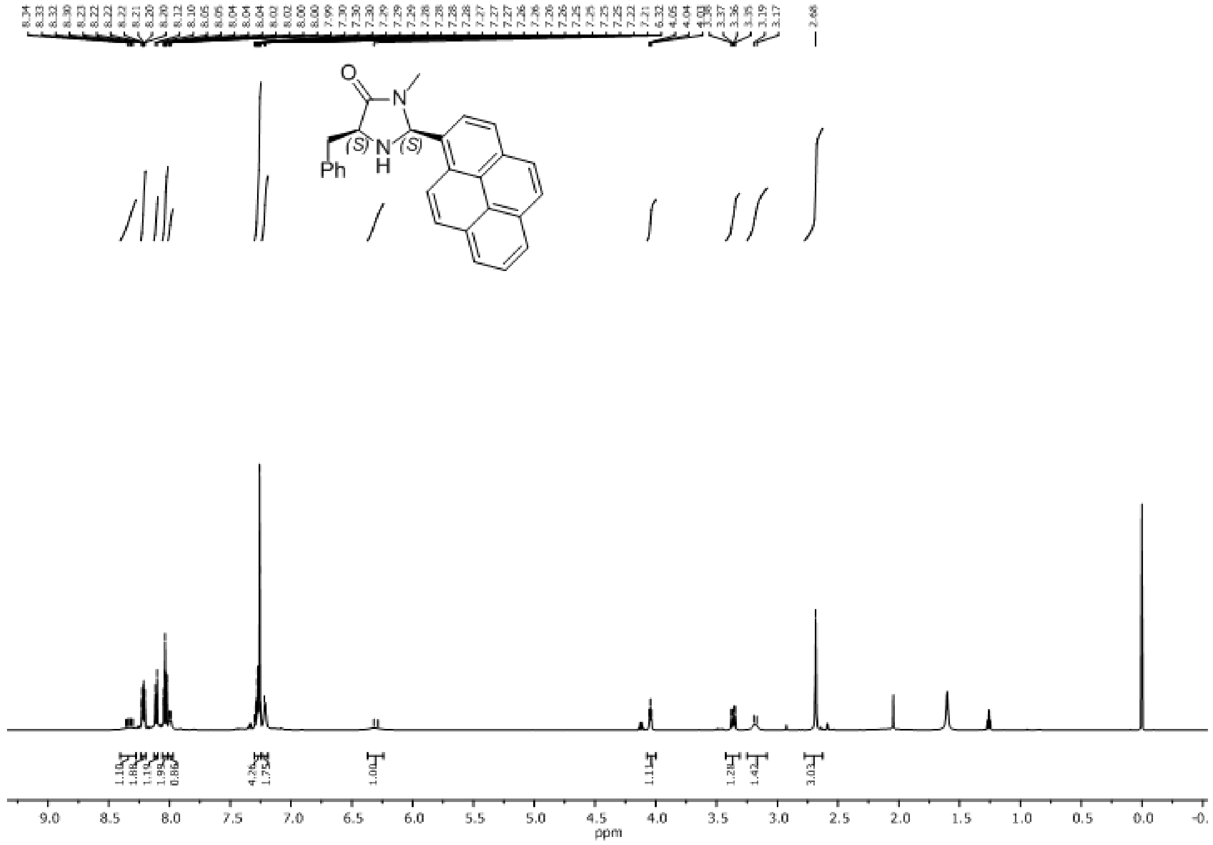

(^{1}H NMR, 600 MHz, $CDCl_3$)

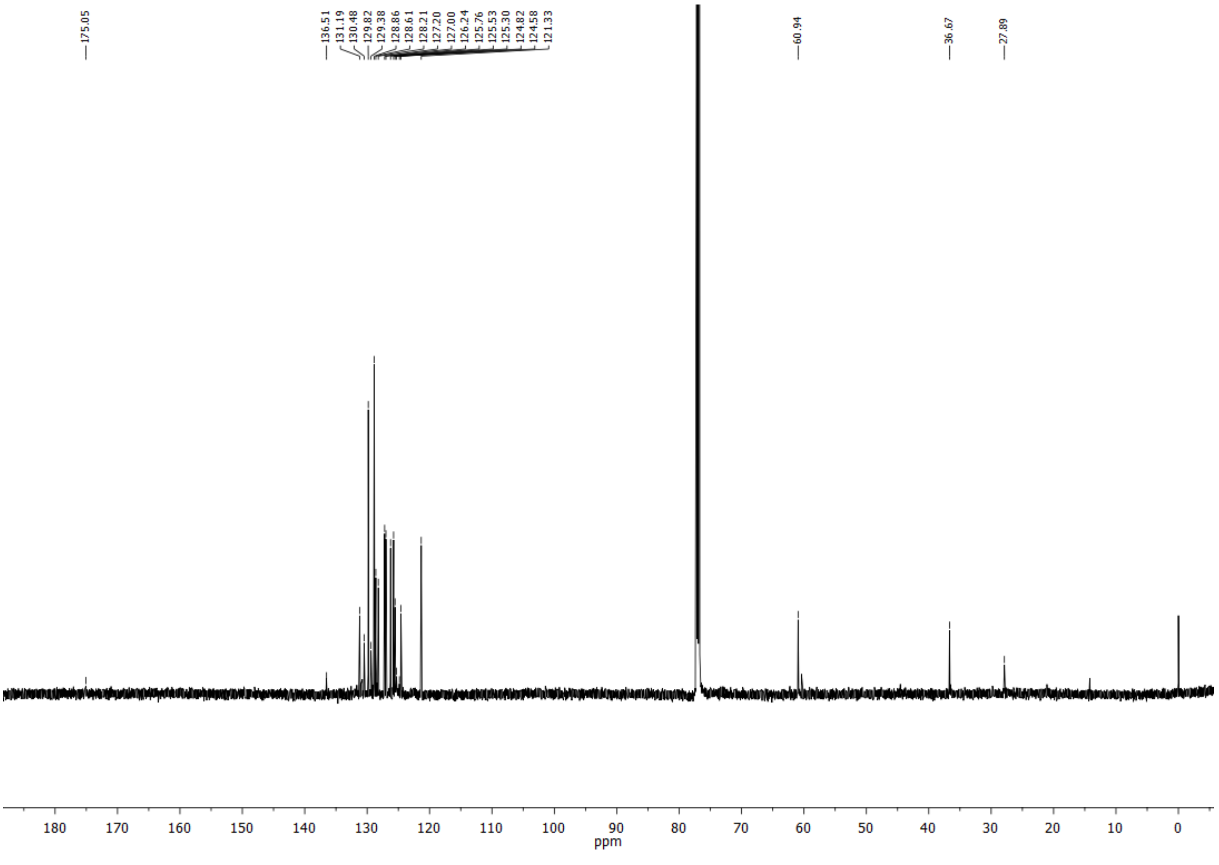

(^{13}C NMR, 150 MHz, $CDCl_3$)

(2*S*,5*S*)-(−)-5-Benzyl-3-methyl-2-mesitylimidazolidin-4-one (3c)

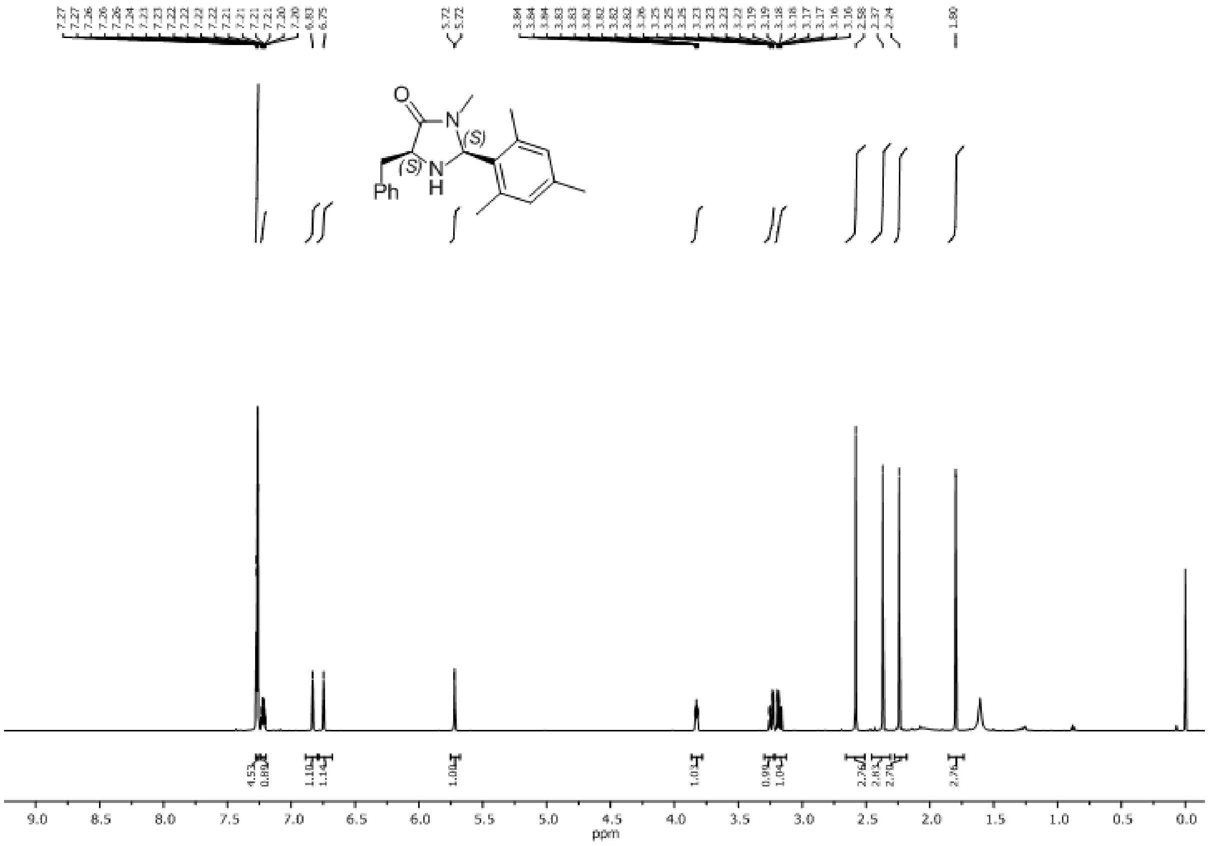

(^{1}H NMR, 600 MHz, $CDCl_3$)

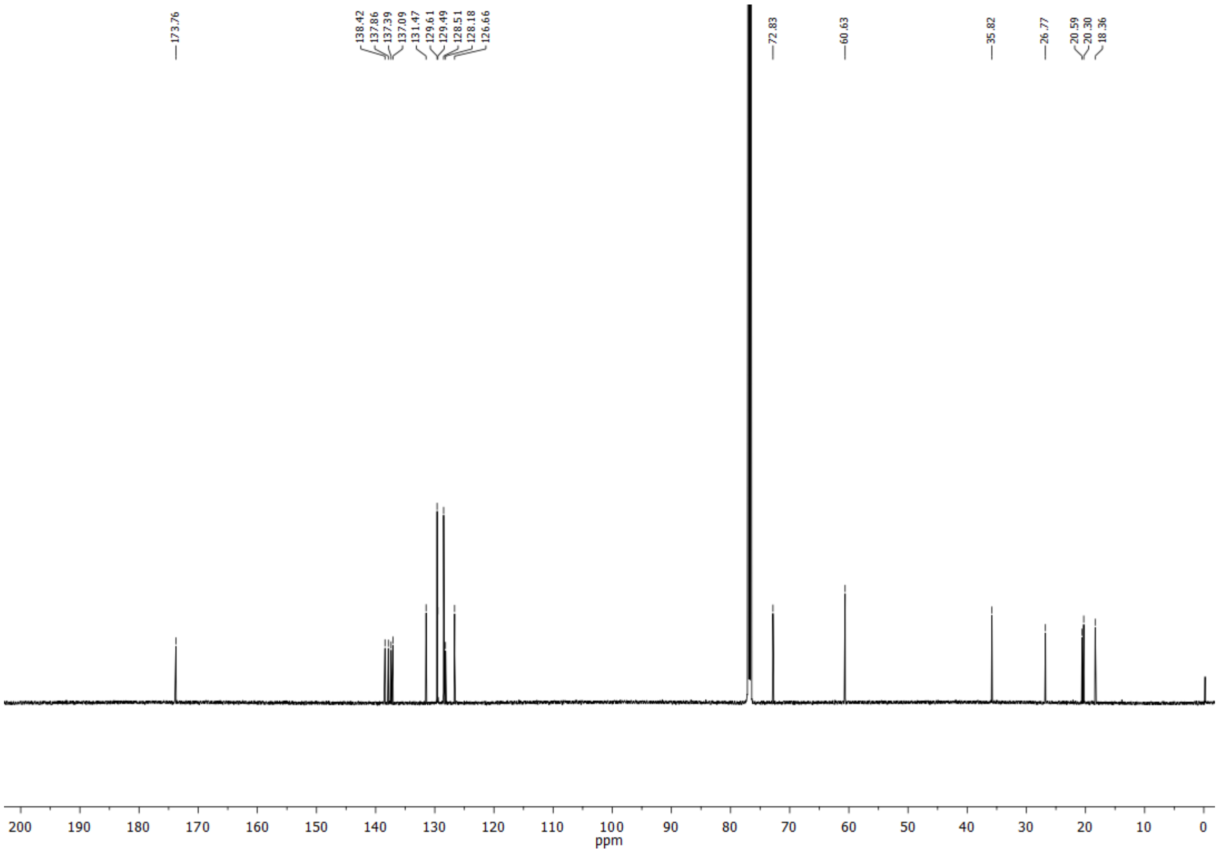

(^{13}C NMR, 150 MHz, $CDCl_3$)

(2*S*,5*S*)-5-Benzyl-3-methyl-2-(2,4,6-triisopropylphen-1-yl)imidazolidin-4-one (3d)

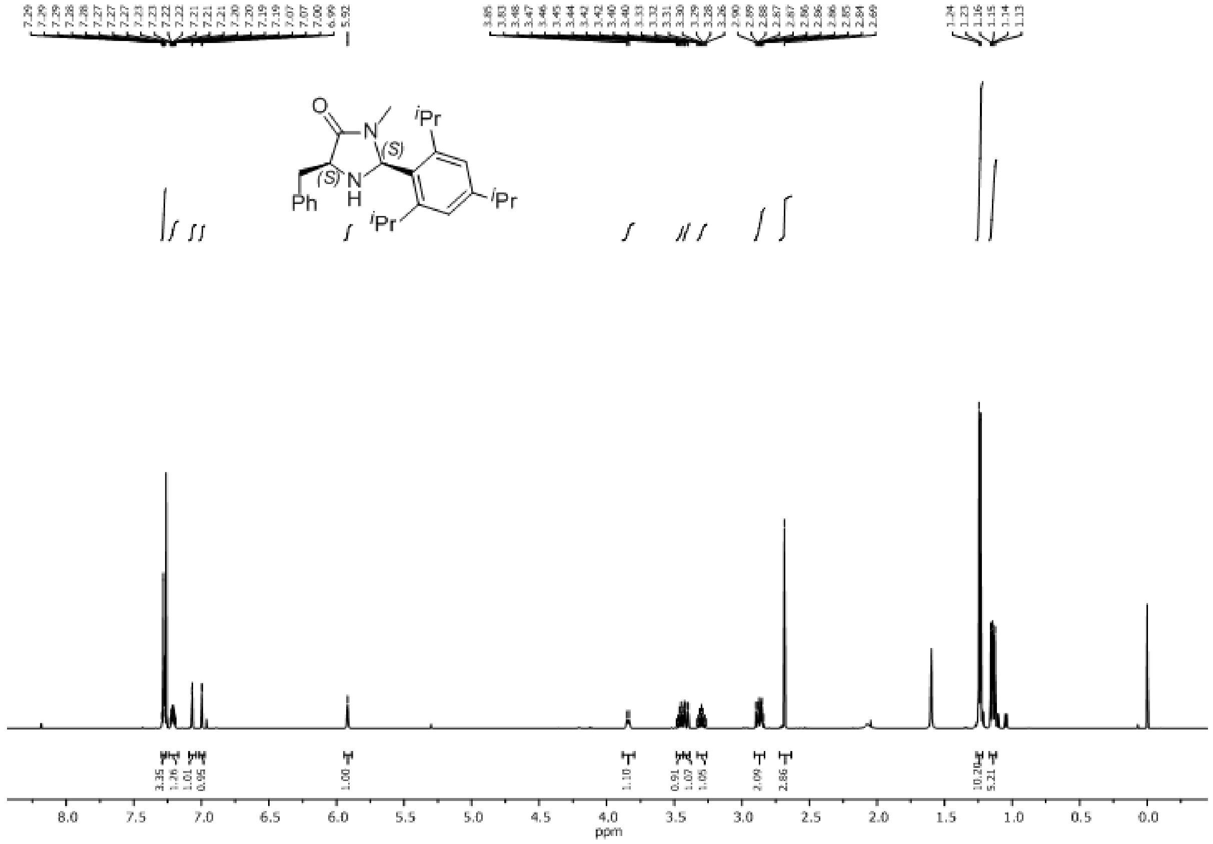

(^{1}H NMR, 600 MHz, $CDCl_3$)

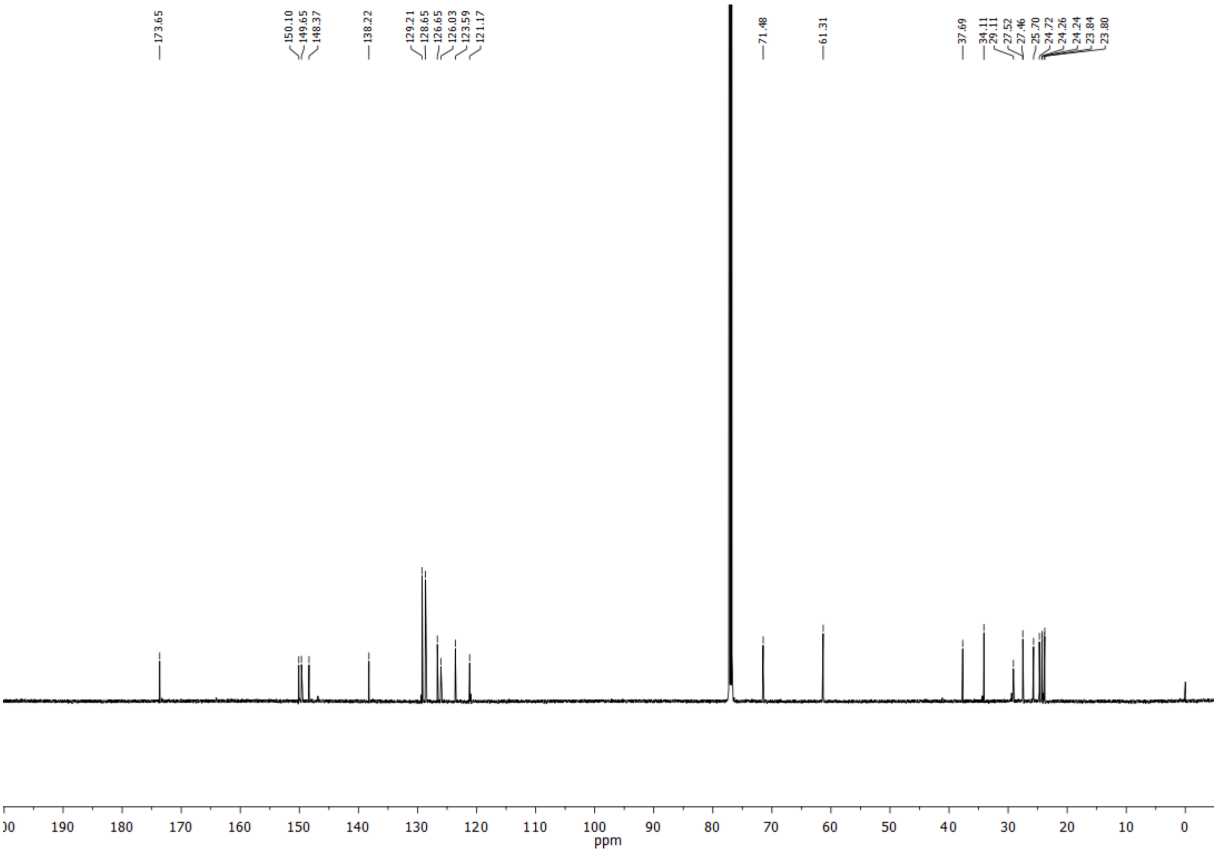

(^{13}C NMR, 150 MHz, $CDCl_3$)
